# Mental Health Issues in Undercover Police Officers: A Systematic Literature Search from a Psychiatric Perspective

**DOI:** 10.3390/healthcare13151933

**Published:** 2025-08-07

**Authors:** Giulia Moretti, Lucrezia Cavagnis, Emma Flutti, Serena Silvestri, Guido Vittorio Travaini

**Affiliations:** 1 Faculty of Medicine, University of Vita-Salute San Raffaele, 20132 Milan, Italy; moretti.giulia@hsr.it (G.M.); emma.flutti@uniroma1.it (E.F.); silvestri.serena@hsr.it (S.S.); travaini.guido@hsr.it (G.V.T.); 2 Department of Human and Social Sciences, University of Bergamo, 24129 Bergamo, Italy; 3 Department of Human Neurosciences, Sapienza University of Rome, 00185 Rome, Italy

**Keywords:** undercover agents, undercover operatives, psychopathological risks, long-term health issues, PTSD, systematic review

## Abstract

**Background:** Undercover police work is a psychologically high-risk profession that exposes officers to chronic stress, identity conflicts, and moral dilemmas. The aim of the present review is to evaluate the psychological consequences associated with undercover police work, focusing on specific psychopathological risk factors. **Methods:** A systematic search was conducted in PubMed, PsycINFO, Web of Science, and Scopus databases. Studies were conducted in the United States, the United Kingdom, New Zealand, and Canada. The present systematic review analyzed data from 380 current undercover operatives, 372 former UCOs, 578 officers without undercover experience, and 60 pre-operational agents. **Results:** From an initial pool of 365 records, 10 studies were identified, of which 6 met the inclusion criteria. The most frequently reported psychological risk factors included anxiety, hypervigilance, identity issues, dissociative symptoms, and substance misuse. These were assessed using validated self-report instruments (e.g., SCL-90), structured interviews, and clinical evaluations. Long-term consequences were more prominent post-deployment, particularly among former UCOs. **Conclusions:** Undercover work is associated with an elevated risk of mental health problems, especially after the end of operations. Future research should focus on standardizing assessment tools and identifying protective factors. The findings support the development of targeted interventions such as pre-deployment psychological screening, ongoing monitoring, and structured reintegration programs to safeguard UCOs’ well-being.

## 1. Introduction

Police work is widely acknowledged as physically and psychologically demanding, with high rates of burnout and suicide risk [1]. Within this challenging field, undercover operatives (UCOs) face a distinctive set of stressors that may exacerbate these risks, making them a particularly vulnerable subgroup within law enforcement. As Girodo [2] defines it, an “undercover law enforcement officer assumes a civic role by posing as a member of a criminal group with a view to gathering intelligence concerning past and or future criminal activities”. The College of Policing [3] further clarifies that “UCOs are deployed under direction in an authorized investigation or operation in which their true identity is concealed from third parties”.

Undercover policing involves officers assuming false identities to infiltrate criminal organizations or gather intelligence. This practice has been utilized globally for decades, with its origins dating back to the 19th century [4]. In recent years, the scope of undercover operations has expanded to address various criminal activities, including drug trafficking, terrorism, human trafficking, and cybercrime [5].

While some intelligence gathering might be passive, such as Closed-Circuit Television (CCTV) recordings or intercepted communications, most undercover work falls under “Human intelligence”, operating alongside Signal Intelligence and Open-Source intelligence. Although popular media often portrays UCOs as heroic figures, the reality is far more complex and challenging. Undercover operations are typically characterized by the following:

Thorough preparation and planning: Officers spend several weeks/months developing their cover identity, studying criminal organizations, and learning specialized knowledge relevant to their assignment.

Strict operational parameters: Contrary to fictional portrayals, UCOs operate within rigid legal and procedural frameworks. They must obtain judicial authorization for their activities, maintain regular contact with supervisors, and adhere to specific operational guidelines that limit their actions and decision-making autonomy.

Varied operational timeframes: Undercover assignments can range from brief encounters lasting hours or days to deep-cover operations extending months or years. Long-term assignments require officers to fully integrate into criminal communities, often necessitating lifestyle changes, relocation, and sustained performance of criminal personas.

Beyond the physical risks, there is a significant psychological distress associated with this profession.

### 1.1. Framework

To comprehensively understand the mental health challenges faced by UCOs, this review adopts a theoretical framework grounded in established models of stress and identity theory. The transactional model of stress and coping developed by Lazarus and Folkman [6] provides the foundational lens through which we examine how UCOs appraise and respond to the specific demands of their work. This model emphasizes the interaction between environmental stressors, individual appraisal processes, and coping responses. Additionally, we integrate identity theory [7] to understand how the constant performance of false identities creates persistent challenges to psychological well-being.

From a psychiatric perspective, we apply stress-vulnerability models that explain how chronic exposure to stressors can precipitate mental health disorders in vulnerable individuals. This framework helps distinguish between normative stress responses and pathological outcomes, guiding our understanding of when intervention becomes necessary.

While psychological research has examined stress in law enforcement, a psychiatric perspective offers distinct advantages in understanding UCO mental health. Unlike general psychological approaches that may focus primarily on performance optimization or basic stress management, psychiatric frameworks emphasize the following: (i) Diagnostic considerations: systematic evaluation of specific mental health disorders prevalent in UCO populations; (ii) Neurobiological impacts: understanding how chronic stress affects brain function and structure; (iii) Treatment implications: informing evidence-based psychiatric interventions tailored to UCO-specific pathology; (iv) Risk stratification: identifying individuals at highest risk for severe psychiatric outcomes.

Research by Violanti and colleagues [1,8] suggests that police work is perhaps the most stressful occupation globally, with officers exposed to more acute and chronic stressors than other professions. This stress exposure is particularly intense for UCOs, who must navigate complex identity issues, switching between their true selves and their criminal personas. Patterson [9] identifies undercover work as the most stressful form of police work, often leading to chronic fatigue, nightmares, and interpersonal conflicts both within and outside their professional environment.

Physical harm and violent retribution also pose significant dangers for undercover officers whose identities might be discovered by criminals. Research shows that the constant fear of exposure creates a persistent state of anxiety and hypervigilance among UCOs [10,11], as maintaining their cover becomes literally a matter of life and death. Adding to these stressors is the frequent necessity for UCOs to participate in criminal activities themselves. In investigating this complex dynamic, Semrad [12] explored the relationship between personality factors and deception capabilities, which are crucial for successful undercover operations. Despite the limited research on UCOs’ deception abilities, Semrad’s analysis revealed that certain traits and skills related to sender demeanor—particularly believability and perceived honesty—appear fundamental to effective deception during undercover work. Complementary evidence by Palena and colleagues [13,14] further underscores the relevance of individual differences, highlighting how personality traits can influence both the tendency to lie and one’s self-perceived competence and attitude toward deception.

UCOs are also at high risk of being shot [10,15], making deception not just a job requirement but a matter of life and death. This chronic stress can lead to various psychological and physiological issues, including cardiovascular problems, immune system suppression, and cognitive impairments [16]. Notably, the anxiety experienced by UCOs does not appear to be linked to pre-existing neuroticism [2], suggesting that it is a direct consequence of the undercover role.

The psychological impact extends beyond immediate stress responses. Research has also indicated an increased risk of substance abuse among UCOs, with a positive correlation between years of experience and the likelihood of alcohol and drug misuse [17,18]. This trend may be attributed to the stress of maintaining a false identity, the need to fit in with criminal elements, or as a maladaptive coping mechanism. The prevalence of substance abuse among UCOs raises concerns not only for their personal health but also for the integrity of undercover operations.

Personality factors such as extroversion, impulsiveness, and narcissism have been associated with higher rates of misconduct and substance abuse in this population, although a highly disciplined self-image appears to mitigate these risks [17].

The psychological toll of undercover work can manifest in various ways, including loss of self-esteem, depression, anger, and a craving for recognition [19]. In some cases, UCOs may experience dissociative symptoms (e.g., depersonalization, derealization, or memory disruptions), maintaining their false identity even outside the context of their undercover work [20]. These symptoms typically arise as a defense mechanism in response to extreme psychological stress and may impair normal identity functioning. Notably, this is particularly concerning when agents have predispositions to dissociative experiences [2].

A particularly concerning aspect of undercover policing is the potential for moral injury. This specific construct refers to the psychological damage that occurs when individuals perpetrate, witness, or fail to prevent acts that violate their fundamental moral beliefs and values. In the context of undercover work, this manifests when officers are required to witness or engage in illegal activities as part of their operations, which can lead to feelings of guilt, shame, and a sense of moral conflict [21]. The prolonged exposure to criminal elements and the necessity of deception can contribute to psychological distress and a loss of self-esteem.

Despite these significant challenges, research on protective factors and effective coping strategies for UCOs remains limited. Although UCOs do attempt to adopt various coping strategies, these are often counterproductive, as evidenced by patterns of alcohol misuse [17,18]. This maladaptive strategy emerges from attempts to self-medicate anxiety, depression, and identity issues but ultimately exacerbates psychological distress and impairs operational effectiveness. Preliminary studies suggest that certain individual characteristics can act as protective factors against stress, not only in the general population but potentially for UCOs as well. These protective traits include resilience, hardiness, self-efficacy, problem-solving skills, internal locus of control, sense of coherence, and strong social support [22].

However, it is important to note that some coping strategies adopted by UCOs may be counterproductive. For instance, emotional detachment, while useful in the short term, may exacerbate long-term psychological issues, including difficulties in emotional regulation and maintaining personal relationships [23]. Indeed, this strategy prevents the natural processing of traumatic experiences and inhibits the development of healthy emotional regulation skills. Research has shown that UCOs with certain personality profiles, particularly those scoring high in extroversion, impulsiveness, and narcissism, are at increased risk of misconduct and substance abuse. Interestingly, when these same traits are coupled with a highly disciplined self-image, the risk of drug and alcohol abuse appears to be lowest within this population [17].

However, many coping strategies adopted by UCOs prove counterproductive, highlighting the urgent need for further research into effective, long-term support mechanisms tailored to the unique demands of undercover work.

### 1.2. The Present Study

The existing literature, while highlighting various stressors and challenges faced by UCOs [9,10,11], lacks a comprehensive synthesis of the psychological sequelae experienced by individuals who engage or have engaged in undercover work. This gap is particularly concerning given the documented high rates of stress, burnout, and potential substance abuse among UCOs [17,18].

Building upon the current state of research, we conducted a systematic review of the literature focusing on the mental health implications of undercover work. Given the relative scarcity of research in this domain and its critical importance for law enforcement effectiveness and personnel wellbeing, our systematic review addresses three fundamental research questions:

1. What is the overall impact of undercover work on officers’ psychological well-being?
2. How does undercover work correlate with specific psychopathological issues?
3. What are the implications of these findings for the selection, supervision, and long-term support of undercover operatives?

For the purposes of this review, we define psychological well-being as encompassing both clinical mental health outcomes (such as anxiety, depression, and PTSD) and broader psycho-emotional functioning (including stress responses, coping mechanisms, and identity issues).

By synthesizing the available literature and addressing these questions, we aim to contribute valuable insights to provide best practices in the management and care of undercover law enforcement personnel, ultimately safeguarding their mental health and enhancing their operational effectiveness.

## 2. Materials and Methods

In conducting the present systematic review, we adhered to the PRISMA (Preferred Reporting Items for Systematic Reviews and Meta-Analyses) guidelines and confirmed compliance by completing the PRISMA checklist [24], available in the Supplementary Materials.

This systematic review was preregistered in the OSF database, https://osf.io/zyphv, accessed on 17 July 2025.

In order to consider studies of comparable quality, the analysis included only peer-reviewed papers published in scientific journals.

The search was conducted on 16 September 2024 in PubMed, PsycINFO, Web of Science, and Scopus online databases, with the last update completed on 26 May 2025.

We adopted an iterative search strategy with the combination of the following terms to capture as many studies as possible: (undercover and (work* or police or agent*) and (health* or symptom* or disorder* or abuse* or trauma* or depress* or anxi* or panic or aggress* or drug* or alcohol*)). Although the interest was focused on mental health-related symptoms, using a more restrictive search string excessively limited the number of potentially relevant articles available. We did not apply any limits or filters.

**Figure 1:**
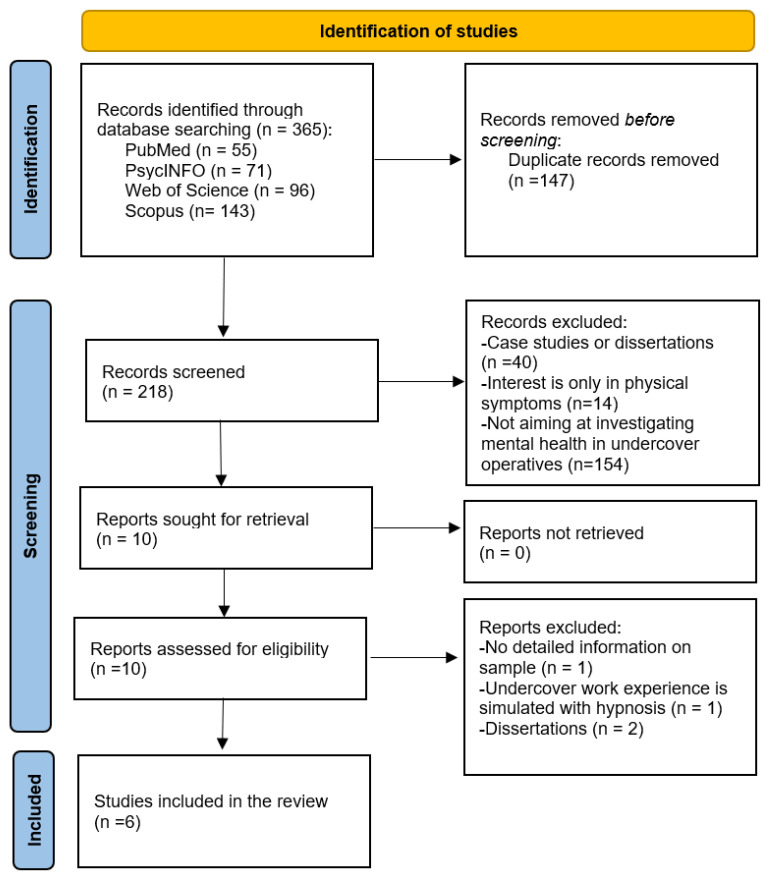
Flowchart for the systematic review procedure.

A total of 365 studies were initially matched. After duplicate records (N = 147) that appeared in each database were removed from the list, 218 articles remained, which were screened for title and abstract. Of these, 10 articles were deemed eligible for full-text review, and they have been analyzed in order to define the final inclusion. At the end of the inclusion process, 6 studies were selected. The process of identifying eligible studies is outlined in Figure 1.

### 2.1. Coding of Studies

The selected studies were read in their full text. Following that, a coding scheme was employed to systematically extract relevant information. For each article, the authors, year, participants’ characteristics, method, and results are reported in Table 1. Further, concerning the implication of UCO’s work on wellbeing, the result section of each article was read in detail, and the statistical estimates were reported in the results section of the article.

Data extraction was conducted using a pre-designed Excel template, specifically developed for this review, to systematically capture relevant study information. The coding process was performed independently by two experienced reviewers, yielding an inter-rater reliability of 90%. Disagreements between reviewers were adjudicated through consultation with a third reviewer, ensuring consensus on all extracted data. The characteristics of the included studies are summarized in Table 1.

### 2.2. Data Synthesis

Given the heterogeneity of study designs and outcome measures, a narrative synthesis approach was employed. The synthesis process involved the following: (i) Thematic categorization: Findings were organized into thematic categories based on mental health outcomes (e.g., anxiety, depression, substance misuse, hypervigilance, dissociative experiences); (ii) Cross-study comparison: Results were systematically compared across studies to identify consistent patterns, contradictory findings, and gaps in the literature.

When quantitative data were available, the following statistical information was extracted and synthesized: Descriptive statistics (e.g., means, standard deviations, and frequencies); inferential statistics (e.g., *p*-values, confidence intervals, and effect sizes); and correlation coefficients (e.g., strength and direction of associations between variables).

### 2.3. Determining Eligibility

Each study included in this review assessed the relationship between undercover work and any symptoms affecting mental health.

Only clinical studies conducted on officers who have/had experience in undercover work have been taken into consideration.

The method used in the six included articles for evaluating the sample depends on the authors (e.g., semi-structured interview, unstructured interview, and symptoms validated checklist) and can be both hetero or self-administered. In some of these studies, the evaluation involved a follow-up and was proposed to the same sample at different times, while in other papers, different groups were compared (e.g., former UC agents vs. present UC agents).

The exclusion criteria for this review are listed below:

- Studies that explored solely physical symptoms related to undercover work;
- Studies that did not report information on sample numbers;
- Studies that simulated (e.g., with hypnosis) undercover work experience;
- Case studies, reviews of existing research, and dissertations related to the topic;
- Articles not written in English.

In most of the included papers, the authors conducted qualitative analyses of their data. However, if reported, the *p*-value has been specified in this review.

### 2.4. Bias Assessment and Heterogeneity

Most of the included studies relied on convenience sampling or voluntary participation, which may limit the generalizability of the findings. Furthermore, there was notable heterogeneity across studies in terms of design (e.g., qualitative vs. quantitative), sample size, participant characteristics (e.g., current vs. former UCOs), and outcome measures. This variability, combined with the small number of available studies, raises concerns about potential publication bias and the underrepresentation of null or negative findings.

However, such limitations are partly inherent to the nature of the population under investigation, as UCOs are typically difficult to access without compromising their anonymity or operational integrity. Additionally, the stigma associated with mental health in law enforcement settings may contribute to underreporting of symptoms.

To mitigate the impact of these biases, we prioritized studies that employed validated psychiatric or psychological assessment instruments, ensured participant anonymity, or adopted standardized data collection procedures. In the synthesis, we qualitatively analyzed findings while explicitly acknowledging methodological differences across studies. Despite the heterogeneity, recurring themes emerged consistently, particularly concerning anxiety, identity disturbance, and substance misuse, suggesting a degree of robustness across different methodological approaches.

### 2.5. Quality Evaluation

The STROBE checklist [29] was used to assess the quality of the six included studies (see Supplementary Materials). This checklist consists of 22 items that should be included in studies such as cohort, case-control, and cross-sectional studies.

It covers key domains such as study design and participants, exposures and outcomes, data sources and measurement methods, risk of bias and statistical analyses, as well as the reporting of results and discussion. STROBE aims to improve the transparency and completeness of reporting in empirical studies, thereby facilitating a clearer understanding of their methodology, findings, and overall validity.

Although no formal threshold guarantees high quality, a higher number of fulfilled items generally reflects better reporting. Among the analyzed studies, only one [27] met 19 out of 22 items, indicating high reporting quality. Two others fulfilled approximately 14 items [19,25], while the remaining three scored lower, at around 12 [28], 13 [15], and 13.5 [25].

Overall, the review suggests medium reporting quality across studies. Consequently, generalizing findings to the wider scientific community may be limited, primarily due to the methodological designs of the included studies.

## 3. Results

Following the systematic search and selection process, six studies were deemed eligible for inclusion in this review (Figure 1).

Study findings were synthesized thematically and are presented across key domains.

### 3.1. Identity Issues

Identity-related issues emerged as a significant concern across studies.

Pogrebin and Poole [26] identified role strain in shifting between the criminal identity at work and the conventional identity at home, with some officers reporting that adopting a deceptive criminal identity for an undercover assignment essentially precluded them from assuming their conventional identity while off duty. Some agents lamented a loss of identity and reported difficulties returning to previous duties, especially due to perceived decreased autonomy.

Macleod [15] noted that anxiety issues were also common and mainly related to identity disturbances, whereas no psychotic episodes emerged during deployment.

Additionally, Curran [19] found that not all undercover agents experienced identity issues, suggesting individual variability in this dimension.

### 3.2. Anxiety, Fear, and Hypervigilance

Anxiety manifestations were consistently documented across studies.

Curran [19] found that the five undercover agents interviewed reported recurring thoughts about the risks associated with their job, which led to fear of being discovered, feelings of being constantly watched or followed, anxiety, hypervigilance, and, for women, fear of being sexually assaulted. The author interpreted these fears as possible indicators of paranoia or paranoid delusion-like symptoms.

Farkas [28] reported that nervous tension affected 39% of officers during deployment, while oversuspiciousness was the most prevalent symptom (44%). Sleep difficulties were reported by 28.3% of officers, and poor concentration by 26.2%. After deployment, oversuspiciousness decreased to 19.5% but remained notable.

### 3.3. Depression and Mood-Related Symptoms

Depressive symptoms showed varying patterns across studies.

Love, Vinson, and Kaufmann [25] found that, compared to current undercovers (N = 254) and officers without undercover experience (N = 578), a greater proportion of former undercover operatives (N = 239) reported the presence of clinical symptoms measured using the Health Concerns Questionnaire (HCQ), including depression, mood changes, and dwelling on problems (all *p*s < 0.05). The results also showed a progressive increase in symptomatology from current UCOs (lowest) to non-undercover officers (moderate) to former UCOs (highest).

Girodo [27] found a significant but negative correlation between depression levels and time spent undercover (*p* < 0.01). In this study, 5 preoperational agents, 9 operational agents, and 10 post-operational agents were categorized as in need of psychiatric services based on SCL-90 total scores. This instrument assesses nine symptom dimensions: somatization, obsessive-compulsive tendencies, interpersonal sensitivity, depression, anxiety, anger-hostility, phobic anxiety, paranoid ideation, and psychoticism. The total SCL-90 score for preoperational agents was significantly lower (*p* < 0.05) than that of both operational and post-operational agents.

### 3.4. Substance Misuse

Alcohol use emerged as a common coping mechanism.

Curran [19] noted that alcohol consumption was frequently used to reduce anxiety, while physical exercise was employed as a more adaptive strategy to cope with stress.

Farkas [28] reported alcohol abuse in 19.5% of officers during deployment, which decreased to 12.5% post-deployment.

Love, Vinson, and Kaufmann [25] found that former undercover operatives reported significantly higher rates of drug use compared to both current UCOs and officers without undercover experience (*p* < 0.05).

### 3.5. Family-Related Difficulties

Love, Vinson, and Kaufmann [25] found that former undercover operatives reported significantly higher rates of marital stress and family problems compared to current UCOs and officers without undercover experience (all *p*s < 0.05).

Farkas [28] identified, through multiple regression analyses, that changes in relationships with family and friends explained 55% of psychiatric symptoms during deployment. Relationship and marital problems affected 27.6% of officers during deployment and 14.2% afterward. Officers working in isolation, rather than within groups, reported significantly greater difficulty maintaining personal relationships and higher levels of loneliness.

Curran [19] noted that distance from family members and the extended duration of assignments contributed to anxiety and stress among UCOs.

Pogrebin and Poole [26] reported that some officers feared for their family’s safety and experienced increased levels of perceived danger due to their undercover work.

### 3.6. Temporal Patterns and Reintegration Difficulties

The timing of symptom emergence showed consistent patterns across studies.

Farkas [28] found that more than half of the officers (52.5%) developed psychological problems during deployment, with symptoms typically emerging around 5.2 months into the assignment. Multiple regression analyses revealed that 55% of psychiatric symptoms were explained by factors such as the inability to discuss the assignment, changes in personal relationships, and the duration of deployment.

Macleod [15] reported that at the end of their deployment, 59% of 37 assessed agents did not report reintegration difficulties, whereas 24% required counselling but successfully re-established psychological health with minor difficulties. However, psychological problems emerged in some cases during follow-ups conducted more than six months later.

Pogrebin and Poole [26] noted that 42% of officers experienced difficulties transitioning to another position following their undercover work.

### 3.7. Risk Perception and Support-Seeking Behaviors

Perceptions of danger and patterns of psychological support-seeking were closely intertwined across the studies. Farkas [28] reported that 26% of officers perceived their lives were in extreme danger during their assignment, while Pogrebin and Poole [26] noted heightened fears for officers’ personal safety and for their families. These high-risk perceptions often coexisted with difficulties in accessing help. Macleod [15] noted that those needing post-deployment assistance were often the most reluctant to initiate therapy. Similarly, 32% of participants in Farkas’s study indicated they would have welcomed psychological support during deployment, underscoring a gap in proactive intervention and engagement strategies.

### 3.8. Positive Outcomes and Comparisons with Control Groups

Despite the prevalence of stressors, several studies identified protective and resilience-related outcomes. Macleod [15] found that 60% of UCOs reported their undercover experience as beneficial, citing outcomes such as personal growth, increased confidence, and satisfaction. Interestingly, the same study hypothesized that moderate levels of non-clinical narcissism may contribute positively to UCOs’ performance.

In terms of behavioral comparisons, Farkas [28] found no significant differences in disciplinary infractions between former undercover officers and a matched control group, suggesting that the undercover experience may not necessarily lead to increased misconduct. Similarly, Girodo [27] reported no significant differences in clinical psychological symptoms between operational and post-operational UCOs, indicating that psychological functioning may remain stable across different stages of deployment.

## 4. Discussion

This systematic review sought to explore the psychopathologies experienced by undercover operatives (UCOs), revealing a complex pattern of psychological risks and challenges that manifest both during and after operational duties. Indeed, working as an undercover is a high-risk job where operators, if discovered, could risk their lives, and the operation could be unsuccessful. Operators frequently face high stressors that can cause burnout or the insurgence of psychopathologies or identity issues.

For example, it was found that UCOs face immediate psychological challenges during active duty, primarily centered around anxiety and hypervigilance. The fear of discovery emerged as a predominant concern, manifesting in what Curran [19] described as paranoid-like symptoms, including persistent feelings of being watched or followed. These findings align with earlier research by Miller [10] and Loftus et al. [11], who identified hypervigilance as a common response to the constant threat of exposure. However, it is noteworthy that these symptoms, while distressing, generally remained subclinical during active deployment, as supported by Macleod’s [15] findings, where 59% of agents maintained operational effectiveness without requiring intervention.

The psychological strain during active deployment appears to emerge from several interrelated sources. The constant management of dual identities, as described by Pogrebin and Poole [26], creates significant cognitive load and emotional strain. This is compounded by the fear of physical harm or discovery, which Macleod [15] identified as a primary stressor. The isolation from support networks, highlighted in Farkas’s work [26], further exacerbates these challenges, with 37% of UCOs reporting significant feelings of loneliness and isolation. Additionally, the moral injury from engaging with criminal activities, a phenomenon explored by Farnsworth et al. [21], creates a particular form of psychological distress that may have long-term implications for moral identity and self-concept.

A striking pattern emerged regarding the timing of serious psychological manifestations. While most UCOs maintained relatively stable mental health during operations, significant psychological difficulties often emerged post-deployment. Love et al. [25] provided compelling evidence for this pattern, demonstrating that former UCOs exhibited higher rates of clinical symptoms compared to both active UCOs and officers without undercover experience. The post-deployment manifestations included PTSD, major depressive disorder, heightened irritability, aggressiveness, substance abuse issues, and persistent identity integration difficulties. This finding is particularly significant when considered alongside Girodo’s research [27], which found that total scores on the SCL-90 test were significantly higher among post-operational agents compared to preoperational agents.

The temporal pattern of symptom emergence suggests that the psychological impact of undercover work may be cumulative and delayed, potentially due to the suppression of psychological distress during active duty when operational effectiveness is prioritized. This aligns with broader research on occupational stress in law enforcement, where Violanti et al. [8] found that chronic exposure to operational stressors can lead to delayed manifestation of psychological symptoms.

### 4.1. Identity Integration

Identity integration and role conflict emerged as particularly significant challenges. Pogrebin and Poole’s [26] findings revealed that UCOs experienced persistent difficulties in transitioning between their criminal and personal identities. This identity strain manifested both during active duty, where officers struggled to maintain their authentic identity during off-duty periods, and post-deployment, where reintegration into conventional police roles proved challenging. These findings are consistent with Girodo et al.’s [20] work on dissociative symptoms in UCOs, suggesting that the psychological mechanisms required for successful undercover work may inadvertently contribute to longer-term identity integration difficulties.

Similar identity conflicts have been documented in other high-risk professions requiring role deception or compartmentalization. Military personnel in deep cover operations [30,31] have shown comparable patterns of identity fragmentation and reintegration difficulties. The phenomenon appears to be particularly pronounced in professions where maintaining false identities is central to operational success, suggesting that identity strain may be an inherent occupational hazard rather than a specific characteristic of law enforcement undercover work.

The relationship between personality factors and operational outcomes proved more complex than initially anticipated. Macleod’s [15] finding that moderate narcissistic traits correlated with positive operational outcomes presents an interesting paradox: traits that might be advantageous during deployment could potentially contribute to adjustment difficulties post-operation. This aligns with French’s [17] research suggesting that certain personality profiles, particularly those high in extroversion and narcissism, may predict both operational success and increased risk for post-deployment difficulties.

### 4.2. Coping Mechanisms and Intervention Strategies

Coping mechanisms employed by UCOs showed varying degrees of effectiveness. Maladaptive strategies, including alcohol use for anxiety reduction and drug misuse, were commonly reported [17,18]. Physical exercise emerged as a more adaptive coping mechanism, though self-reflection, while potentially beneficial, sometimes led to increased guilt as noted by Curran [18]. The relatively high rates of substance abuse among former UCOs, as documented by Love et al. [25], suggest that maladaptive coping strategies may persist well beyond the active deployment period.

Drawing from interventions developed for similar high-risk professions, several promising approaches warrant consideration. Cognitive-behavioral therapy (CBT) interventions specifically adapted for identity integration issues have shown effectiveness in military populations [32]. Eye Movement Desensitization and Reprocessing (EMDR) has demonstrated efficacy in treating PTSD symptoms in military members [33]. Additionally, peer support programs, such as those implemented for veterans, have shown promise in reducing isolation and improving community integration [34].

Mindfulness-based interventions have been successfully adapted for personnel requiring role flexibility in high-stress environments [35]. These interventions focus on developing healthy psychological boundaries between professional and personal identities, which appear particularly relevant for UCOs experiencing identity integration difficulties.

### 4.3. Cultural and Geographic Influence

The geographic scope of existing literature reveals important cultural considerations that may influence psychological outcomes and coping strategies. The reviewed studies primarily originated from Western countries (the United States, the United Kingdom, New Zealand, and Canada), representing individualistic cultures with specific law enforcement traditions and support systems.

Cultural factors may significantly influence how UCOs experience and cope with psychological stressors. In collectivistic cultures, the isolation from family and community support networks identified by Farkas [28] may be particularly devastating, given the central role of social connections in psychological well-being.

The stigma associated with mental health help-seeking, which varies significantly across cultures, may influence UCOs’ willingness to engage with psychological support services. Macleod’s [15] finding that agents requiring assistance were reluctant to initiate therapy may be particularly pronounced in cultures where mental health stigma is more severe or where law enforcement culture emphasizes self-reliance.

Different cultural approaches to moral injury and ethical conflicts may also influence how UCOs process the psychological impact of engaging with criminal activities.

### 4.4. Long-Term Mental Health Trajectories

The long-term impact of undercover work on mental health extends well beyond immediate post-deployment difficulties. While the reviewed studies provided limited data on post-retirement outcomes, the available evidence suggests that psychological effects may persist for decades after active service.

The identity integration difficulties documented during active duty and immediate post-deployment periods may contribute to ongoing psychological challenges throughout UCOs’ careers and into retirement. The compartmentalization skills developed during undercover work, while operationally necessary, may interfere with the integration of life experiences that typically occur during later life stages and retirement transitions.

Post-retirement mental health trajectories in UCOs may be complicated by several factors related to their operational experience. The inability to discuss significant proportions of their career due to confidentiality requirements may impair the life review processes that typically facilitate healthy aging [36].

Furthermore, the delayed manifestation of psychological symptoms documented in the reviewed studies suggests that some UCOs may not experience the full impact of their operational exposure until years after retirement, when natural age-related changes in coping resources may compound existing vulnerabilities. Consequently, long-term monitoring and support programs that extend beyond active service periods are necessary.

Farkas’ [28] comprehensive analysis provided crucial insights into risk factors for psychological distress. Working in isolation, as opposed to group operations, significantly increased the likelihood of psychological difficulties. The limited ability to discuss assignments, combined with extended deployment durations, created an additional psychological burden. Perhaps most significantly, the study identified that psychological symptoms typically emerged approximately five months into deployment, providing an important timeline for intervention planning.

Clinical implications of these findings are substantial and multi-faceted. The need for proactive psychological monitoring becomes evident, particularly during the identified risk period around five months into deployment. The development of targeted interventions for post-deployment transition periods appears crucial, given the high rates of psychological difficulties among former UCOs. Regular psychological screening for former UCOs, as suggested by Love et al. [25], could help identify and address emerging psychological issues before they become severe.

### 4.5. Limitations and Future Directions

It is important to note that this systematic review has some limitations. First, the available literature on the topic is scarce; thus, database searches yielded a limited number of records, mostly from a few countries (the United States, the United Kingdom, New Zealand, and Canada). This can, of course, be due to the topic itself: Undercover operations are inherently high-risk and require strict confidentiality regarding the identities of the officers involved, including their gender, age, and other sociodemographic information that could potentially reveal their true identity. Nonetheless, collaborations between academic institutions and law enforcement agencies could be established to study this phenomenon while preserving the anonymity of UCOs. It is also plausible that additional data exists but remains unpublished.

Second, the inconsistent methods used to analyze psychopathological risks hinder solid conclusions. Standardizing research methods, outcomes, and variables would facilitate more robust comparisons. Specific methodological improvements should include the following: (i) Implementation of standardized assessment protocols specifically validated for UCO populations, such as modified versions of the PTSD Checklist for DSM-5 (PCL-5) that account for the unique stressors of undercover work; (ii) Development of UCO-specific measures for identity integration difficulties and moral injury; (iii) Adoption of longitudinal designs with standardized follow-up intervals (e.g., 6 months, 1 year, 2 years, 5 years post-deployment); (iv) Integration of biomarker assessments (cortisol, inflammatory markers) to complement self-report measures; (v) Implementation of cross-cultural validation studies to ensure assessment tools are appropriate across different cultural contexts.

Third, the variation in investigated symptoms makes it difficult to identify the primary psychological impacts of undercover work. Future research should aim at validating standardized assessment protocols specific to UCOs, evaluating various intervention strategies, and examining the long-term psychological trajectories of UCOs, especially post-retirement. Additionally, cross-cultural studies on how different agencies address UCO psychological support could provide useful insights for establishing best practices.

Future research priorities should include the following: (i) Prospective longitudinal studies following UCOs from pre-deployment through retirement; (ii) Randomized controlled trials of intervention strategies specifically designed for UCO populations; (iii) Cross-cultural comparative studies examining how different legal systems and cultural contexts influence UCO psychological wellbeing; (iv) Development and testing of family-based interventions to address the impact of UCO work on family systems; (v) Studies examining the effectiveness of peer support programs and their optimal implementation; (vi) Investigation of the role of organizational factors (agency culture, supervision quality, and workload) in UCO psychological outcomes.

Furthermore, it is essential to consider that, while the STROBE checklist is a valuable tool for assessing the quality of the included studies, it is not sufficient on its own for a comprehensive systematic review since it does not cover all aspects of the studies’ quality. As a tool to estimate the completeness and clarity of reporting, it should be used in conjunction with other quality assessment tools and critical thinking to make a comprehensive evaluation.

Although our review includes only a limited number of studies, it currently stands as the only available synthesis specifically examining the mental health of UCOs. Given this substantial gap in existing literature, future research should prioritize more rigorous methodologies, such as randomized controlled trials (RCTs) and experimental studies.

The present systematic review serves as a foundational step, providing initial insights into the potential psychological implications of this unique profession. Given the significant psychological impact of undercover work, it is crucial to implement comprehensive support systems for UCOs. Pre-employment screening and ongoing psychological monitoring can help identify and address potential issues at an early stage.

Interventions implemented both during and after deployments, together with regular post-deployment screenings, can help mitigate long-term psychological difficulties and promote overall well-being. Notably, the high-risk period, approximately five months into deployment, highlights the need for targeted monitoring and customized post-deployment support, which may significantly reduce psychological problems among former UCOs. The development of comprehensive, culturally sensitive, and long-term support systems represents a critical priority for law enforcement agencies worldwide.

## 5. Conclusions

This systematic review underscores the urgent need to re-evaluate the selection, training, support, and reintegration processes for undercover operatives (UCOs). The findings present significant implications for law enforcement agencies and mental health professionals involved with this vulnerable population.

The existing literature reveals distinct categories of psychological issues that UCOs face. During active deployment, UCOs primarily experience anxiety-related symptoms and hypervigilance, with paranoid-like symptoms manifesting as persistent feelings of being watched or followed. Quantitative data shows that 37% of UCOs report significant feelings of loneliness and isolation during operations, while 59% maintain operational effectiveness without requiring immediate intervention.

Post-deployment manifestations are more severe and include PTSD, major depressive disorder with heightened irritability and aggressiveness, substance abuse issues (particularly alcohol misuse for anxiety reduction), and persistent identity integration difficulties.

Notably, personality traits play a crucial role in UCO selection and performance. As highlighted by Macleod [15], moderate levels of narcissism are linked to positive operational outcomes, while excessive narcissism may pose risks, indicating a need for personality assessments. This complex relationship between personality traits and effectiveness suggests that intelligence services should implement sophisticated psychological screening protocols that go beyond inclusion/exclusion criteria.

Identity management abilities also emerge as essential for UCOs, not only during initial selection but as an ongoing area of focus. Specific assessments and training in identity flexibility are needed throughout a UCO’s career to support adaptability. This can be achieved through structured simulations, regular psychological evaluations, and evidence-based training protocols, such as role-playing exercises and virtual reality simulations, focusing on initial assessment of identity flexibility and resilience, regular monitoring of identity integration during operations, and structured support for identity reintegration after deployment.

Further, the development of adaptive coping strategies is paramount. Given the high prevalence of maladaptive coping methods, particularly alcohol misuse [17,18], proactive intervention is essential. Health-promoting interventions should aim to develop protective factors, including building resilience through structured programs, enhancing hardiness with exposure-based exercises, strengthening self-efficacy through graduated challenges, and promoting physical fitness as a positive coping mechanism.

The psychological impact of undercover work extends well beyond immediate post-deployment difficulties, with evidence suggesting effects may persist for decades after active service. Indeed, post-retirement mental health trajectories may be complicated by the inability to discuss significant career portions due to confidentiality requirements, potentially impairing life review processes that facilitate healthy aging.

Most critically, mandatory psychological support is essential throughout an undercover agent’s career. Evidence shows that psychological issues frequently emerge post-deployment [25], suggesting that long-term monitoring and support are imperative. Due to reluctance among UCOs to seek support despite experiencing symptoms, agencies should implement mandatory psychological screening at regular intervals, structured debriefing protocols post-operation, comprehensive assessments before reintegration into regular duties, long-term follow-up protocols for former UCOs, and the development of therapeutic approaches tailored to UCOs.

The present systematic review identifies significant gaps in understanding how gender and the type of undercover work influence psychological outcomes. The geographic scope of existing literature, primarily from Western individualistic cultures (United States, United Kingdom, New Zealand, and Canada), limits generalizability. Moreover, gender-specific considerations remain largely unexplored. Similarly, variations in psychological impact based on operation type (drug enforcement, organized crime, terrorism) require further investigation.

There is also a need for better integration between intelligence services and territorial psychiatric services to ensure continuity of care, with strict confidentiality and security. Standardized protocols for secure communication and information sharing would enhance treatment outcomes. This could involve the development of secure communication channels, standardized assessment and reporting protocols, clear guidelines for information sharing that respect operational security, and emergency response protocols for acute psychological crises.

In conclusion, supporting the psychological well-being of UCOs is not only an individual health priority but also a public safety concern. Compromised psychological functioning in UCOs can affect operational effectiveness, decision-making capabilities, and mission success rates. The delayed manifestation of psychological symptoms means that untreated post-deployment difficulties may impact UCOs’ subsequent law enforcement roles, potentially affecting public safety outcomes. Effective selection, support, and monitoring of these operatives are crucial for their well-being and the success of law enforcement missions. As our understanding of UCOs’ psychological challenges evolves, so must our strategies to address their mental health needs. This requires ongoing collaboration between law enforcement, mental health professionals, and researchers to develop and implement evidence-based protocols for UCO selection, training, support, and reintegration.

## Figures and Tables

**Table 1:** Characteristics of studies included in the systematic review.

Title, Author(s) and Year	Sample	Method	Results
Undercover policing: a Psychiatrist’s Perspective Macleod, 1995. [15]	Volunteers were selected from an initial pool of 37 officers with several years of police service. The New Zealand police department preferred not to disclose the precise number of undercover agents. 81% male. 62% were single. 22% had prior military experience. Undercover agents M_age_ = 25.4. None with a prior psychiatric history. Mean length of police service 4.5.	Psychiatric assessment (opinions, ideas, clinical assessment, and evaluation) of the author about UC agents, from selection through deployment and end of the commitment. Individual and group psychological debriefing at the end of the operation.	Narcissistic traits were exacerbated during deployment. Subjective view of the psychiatrist was that moderate narcissism was associated with positive operational outcomes. Minor raises of anxiety were common and related to identity issues. 16% undercover agents suffered major psychological or psychiatric sequelae, such as PTSD, major depressive disorder, difficulties regaining their prior police identity, and lifestyle. 60% felt they obtained positive gains from deployment, such as satisfaction and personal growth.
An Exploration of Well-being in Former Covert and Undercover Police Officers Curran, 2020. [19]	5 former English undercover police officers. 3 males, 2 females. Psychiatric history was not provided. M_age_ of the UC officers and the mean length of police service were not specified.	Semi-structured interviews were recorded (questions were developed from undercover policing literature specifically to look at identity stressors). Basic interview protocol was developed; subsequently, depending on the participants’ responses to these questions, ad-hoc follow-up questions helped to draw out and expand upon the salient aspects of each officer’s experience. Thematic analysis.	Fear of being discovered (3). Feeling like they were being watched or followed (1). Anxiety and hypervigilance (2). Fears of being sexually assaulted (women) (2). Alcohol is associated with the need to reduce feelings of anxiety.
Symptoms of Undercover Police Officers: A Comparison of Officers Currently, Formerly, and Without Undercover Experience Love, Vinson, Tolsma & Kauffmann, 2008. [25]	254 current undercover officers, 93% male, M_age_ 36.2. 578 officers without undercover experience, 92% male, M_age_ 34.9. 239 former undercover officers,94% male, M_age_ 38.6. Psychiatric history was not provided. Mean length of the Michigan (USA) police service was not specified.	Health Concerns Questionnaire (54 psychological and physical symptoms; self-report instrument).	The HCQ investigated 54 symptoms divided into 4 clusters: (1) depressed/psychosocial symptoms include low energy, poor sleep, poor concentration; (2) somatization-dominant symptoms like pain, muscle weakness, worry about health; (3) broad/severe symptoms: such as depression, worthlessness, memory problems, irritability; (4) mild complaint symptoms: e.g., inactivity, headaches, muscle tightness. Former UC officers reported significantly higher frequencies of almost all clinical symptoms than both current UC officers and officers without UC experience. Increased frequencies of symptomatology from current UC officers (lowest) to officers without UC experience (higher), to former UC officers (highest).
Vice isn’t nice: a look at the effects of working undercover Pogrebin & Poole, 1993. [26]	20 present and 20 former undercover officers representing three federal law enforcement agencies and eight municipal police departments located in the greater Denver metropolitan area (USA). 35 men and 5 women. Their ages ranged from 28 to 45 (mean = 37), and their undercover experience ranged from 1 to 7 years (mean = 4). Psychiatric history was not provided.	Unstructured interviews. All interviews were conducted at the respective agencies in either subject offices, private conference rooms, or interrogation rooms. Each interview lasted approximately two hours and was tape-recorded with the subject’s consent. The interview tapes were subsequently transcribed for qualitative data analysis.	Increased risk of stress-induced illness, physical harm, or corruption. Federal agents were found to experience profound changes in their value systems, often resulting in overidentification with criminals. Constant tension between loyalty and betrayal in performing an undercover role. 2 UC officers felt morally tainted by the undercover experience. Some UC officers experience role strain in shifting between the criminal identity at work and the conventional identity at home. For some officers, adopting a deceptive criminal identity for an undercover assignment essentially precludes their assuming their conventional identity while off-duty. UC officers worry about the safety of their families when they are with them in public. Heightening feelings of uncertainty and danger associated with the work. Loss of identity. 2 former UC officers: issues related to coming back to previous duties, decreased autonomy, and personal initiative.
Symptomatic Reactions to Undercover Work Girodo, 1991. [27]	Undercover agents (150 male, 5 female) groups from Canada: 1. Preoperational agents (60) who had completed undercover training but had yet to assume their first assignment. M_age_ = 29. 2. Operational agents (35) who were currently in the middle of an undercover operation. M_age_ = 31. 3. Post operational agents (60) who had recently completed an undercover investigation and who had returned to other duties. M_age_ = 32. Psychiatric history was not provided.	Two psychiatric survey instruments: 1. HOS (Health opinion survey), which is composed of 20 items. It provides a measure of global reactions in responding to temporary stressors. A critical score can discriminate between those who would be identified as psychiatrically at risk and those who are not. 2. SCL-90 (Symptom Checklist). It identifies 9 symptom dimensions of moderate to severe psychological disturbance as experienced over the previous week (90 items).	Five preoperational agents, 9 operational agents, and 10 post-operational agents would be categorized as in need of psychiatric services. The scores for at-risk agents were associated with operational status (preoperational, operational, post-operational). Preoperational scores (M = 41.9) significantly differed from those of operational (M = 103.2) and post-operational (M = 82.7). Higher incidence of mental disturbance and more severe psychiatric symptoms among active vs. inactive undercover agents.
Stress in Undercover Policing Farkas, 1986. [28]	82 current and former undercover (UC) officers from Honolulu Police Department: 68 former UC officers are still employed 9 current UC officers 5 former UC officers no longer with Department Mean age: 26.2 years (range 20–42 years) Assignment duration: 1–48 months (mean = 13.3 months) Only 7.3% attended police recruit school prior to UC assignment Psychiatric history was not provided.	Survey of 121 items related to stress in undercover operations. Assessment included: Mental status before, during, and after assignment Lifestyle changes Perceptions of departmental support Attitude changes Demographic information Administration varied by group. Most former officers: Group administration in classroom. Current UC officers: Individual interviews at off-site locations. Former officers no longer with department: Mailed surveys. Second experiment compared UC officers with matched control group for rule infractions	52.5% reported psychological problems, emerging 5.2 months into assignment. Most common symptoms during UC work: Oversuspiciousness (44%) Nervous tension (39%) Loneliness/isolation (37%) Sleep difficulties (28.3%) Relationship/marital problems (27.6%) Stress factors: Inability to talk about assignments Negative changes in relationships Isolation. 42% reported transition problems after assignment 32% indicated need for psychological support during assignment. Training and support issues: 56% reported inadequate preparation/training 25% reported inadequate supervision 29% reported receiving no information about assignment nature beforehand. Criminal association impacts: 16% “frequently” developed friendships with criminals 39% “occasionally” developed such relationships 9% reported “frequently” engaging in criminal activity 7% reported “occasionally” engaging in criminal activity.

## Data Availability

Not applicable.

## Supplementary Materials

The following supporting information can be downloaded at: https://www.mdpi.com/article/10.3390/healthcare13151933/s1, Document S1: Strobe checklist; Document S2: PRISMA checklist.

## Abbreviations

The following abbreviations are used in this manuscript:

PTSD	Post-traumatic stress disorder
UCO	Undercover operative
